# Editorial: The role of physical education in adolescent life satisfaction and well-being

**DOI:** 10.3389/fspor.2026.1901270

**Published:** 2026-06-29

**Authors:** Marco Batista, Carmen Galán-Arroyo, Antonio Castillo-Paredes, Jorge Rojo-Ramos

**Affiliations:** 1 Polytechnic University of Castelo Branco, Castelo Branco, Portugal; 2 SPRINT—IPCB, Sport Physical Activity and Health Research & Innovation Center, Castelo Branco, Portugal; 3 Promoting a Healthy Society Research Group (PHeSO), Faculty of Sport Sciences, University of Extremadura, Cáceres, Spain; 4 Grupo AFySE, Investigación en Actividad Física y Salud Escolar, Escuela de Pedagogía en Educación Física, Facultad de Educación, Universidad de Las Américas, Santiago, Chile

**Keywords:** adolescent, life satisfaction, motivation, physical education, well-being

## Introduction

Adolescence is a critical period of human development, characterised by profound biological, psychological, social and behavioural changes that influence health, well-being and quality of life across the lifespan. In this context, the promotion of subjective well-being and life satisfaction has gained increasing prominence in international research and is recognised as an important indicator of positive development, social adjustment and mental health (1). At the same time, insufficient levels of physical activity observed among young people remain a global public-health concern, with the World Health Organization (2) warning about the high prevalence of sedentary behaviours during adolescence.

Scientific evidence consistently shows that regular participation in physical activity is associated with significant physical, psychological and social benefits, including better mental health, higher self-esteem, lower levels of anxiety and depression, and greater subjective well-being (3, 4). Within this framework, Physical Education occupies a privileged position as an educational context capable of promoting not only motor skills but also the personal, social and emotional development of young people (5).

Contemporary perspectives also underscore the relevance of the concept of Physical Literacy, understood as the combination of motivation, confidence, physical competence, knowledge and understanding that enables individuals to value and take responsibility for participating in physical activities throughout life (6). Likewise, Self-Determination Theory suggests that educational contexts that support autonomy, competence and interpersonal relatedness tend to foster more self-determined forms of motivation and higher levels of well-being (7).

It was in this context that the Research Topic *The Role of Physical Education in Adolescent Life Satisfaction and Well-being* emerged. The eleven articles included in this collection offer a comprehensive perspective on how Physical Education, physical activity, Physical Literacy, family and institutional contexts, and various psychological mechanisms contribute to the promotion of well-being among adolescents and young adults. Despite thematic and methodological diversity, the studies converge on a common message: the effects of Physical Education and physical activity on life satisfaction and well-being are explained by a complex network of motivational, psychological, social and contextual mechanisms.

## Physical education, physical literacy and motivation as foundations of well-being

One of the main contributions of this special issue is to demonstrate that Physical Education plays a role that far exceeds the mere promotion of physical activity within the school setting. The studies published suggest that Physical Education can constitute an important mechanism for developing motivation, Physical Literacy and sustained engagement in physically active behaviours.

The study by Batista et al. is probably the work most directly aligned with the central theme of this collection. The authors showed that adolescents who attribute greater importance to Physical Education exhibit higher levels of positive affect, greater life satisfaction and stronger intentions to remain physically active. Particularly relevant was the identification of the intention to be physically active as a mediating variable in the relationship between the importance attributed to Physical Education and subjective well-being. These results suggest that the influence of Physical Education is not limited to participation during lessons, but contributes to the construction of behavioural orientations that may support active lifestyles across the lifespan.

The importance of motivation and sustained engagement is also evidenced by the scope review conducted by Bertills et al., which analysed school-based interventions targeting sedentary adolescents. The findings indicate that programmes implemented within Physical Education classes can increase physical activity levels when supported by motivational strategies, self-regulation processes and individualised teacher support. These results reinforce the idea that the quality of experiences in Physical Education plays a decisive role in promoting active behaviours.

Physical Literacy emerges as another central element in this collection. Mendoza-Muñoz et al. demonstrated that perceived levels of Physical Literacy vary significantly according to sociodemographic factors. Girls showed lower values than boys, while adolescents from more advantaged socioeconomic backgrounds and with more highly educated mothers revealed higher levels of Physical Literacy. These findings highlight that opportunities to develop confidence, competence and motivation for physical activity are not distributed equitably.

In turn, Harbichová et al., through the psychometric validation of the Czech version of the PLOC-R, reinforced the relevance of Self-Determination Theory for understanding young people's behaviours in the Physical Education context. The results showed that more autonomous forms of motivation are positively associated with perceived competence, autonomy, interpersonal relatedness and engagement in lessons.

Finally, Alayan et al. showed that, although students attribute high importance to Physical Education, the existence of institutional constraints — including limited contact hours, frequent class cancellations and a scarcity of extracurricular opportunities — can undermine the subject's potential to promote active lifestyles.

Taken together, these studies suggest that Physical Education influences well-being not only through physical participation, but above all through the promotion of motivation, Physical Literacy and the intention to remain active throughout life.

## Physical activity, mental health and psychosocial well-being

Another central axis of this collection concerns the mechanisms through which physical activity contributes to mental health and psychological well-being. Rather than merely demonstrating direct associations between physical activity and mental-health indicators, several studies explored the mediating processes that explain these relationships.

Hu et al. found that physical activity is negatively associated with negative emotions among adolescents, and also showed that a substantial part of this effect is explained by social competence and the quality of interpersonal relationships. The results suggest that physical activity contributes to reductions in anxiety and depression not only through biological mechanisms, but also because it provides opportunities to develop social skills, strengthen relationships and increase the sense of belonging.

This interpretation is reinforced by the findings of Liu et al., who examined the impact of rituals associated with sporting events on the well-being of university students. The authors demonstrated that the benefits of these experiences are partially explained by increased perceived social support. Thus, sport emerges as a social phenomenon capable of promoting collective identity, social integration and psychological well-being.

Similarly, Fu and Huang identified the experience of flow as a mediator of the relationship between physical activity, subjective well-being and depressive symptoms. The results suggest that the psychological benefits of physical activity are enhanced when individuals experience high levels of engagement, concentration and enjoyment during practice.

A particularly innovative perspective was presented by Cao and Han, who showed that physical activity is negatively associated with the phenomenon of “lying flat”, characterised by demotivation, withdrawal and disengagement from social demands. The authors found that this effect is explained by a mediating chain involving future-oriented temporal focus and a sense of meaning in life. These findings suggest that physical activity can contribute to the development of personal goals, existential purpose and active engagement with life.

Together, these studies demonstrate that the benefits of physical activity for well-being are largely explained by psychosocial mechanisms related to interpersonal relationships, social support, positive experiences, motivation and the construction of meaning.

## The importance of family, social and institutional contexts

One of the most consistent conclusions of this collection is that physical activity and well-being do not depend exclusively on young people's individual characteristics. Family, social and institutional contexts also play a decisive role.

Xie et al. showed that different forms of family capital significantly influence adolescents' participation in physical activities. Maternal education, household income, parents' health status and the quality of family social networks emerged as important predictors of physical-activity behaviours. These findings reinforce the idea that opportunities to engage in physical activity are strongly conditioned by the resources available within the family context.

Converging evidence from Mendoza-Muñoz et al. found that socioeconomic and family factors significantly affect perceived levels of Physical Literacy. The repeated importance of maternal education across both studies suggests that the family environment plays a particularly important role in shaping attitudes, skills and behaviours related to physical activity.

The results reported by Alayan et al. add an institutional dimension to this discussion. Despite students' positive attitudes towards Physical Education, the existence of structural constraints substantially reduces participation opportunities. This evidence suggests that the transformative potential of Physical Education depends not only on students' motivation but also on educational institutions' capacity to provide favourable conditions for practice.

Taken together, the studies gathered in this collection underscore the need to adopt an ecological perspective on the promotion of physical activity and well-being, recognising the simultaneous influence of individual, family, school and social contexts.

## Future perspectives

Despite the advances provided by the studies included in this special issue, important challenges remain for future research. First, the predominance of cross-sectional designs limits our understanding of the causal relationships between Physical Education, physical activity and well-being. Longitudinal studies will be essential to better characterise these trajectories across development.

Second, the findings highlight the need to deepen research on the mediating mechanisms responsible for the observed benefits. Variables such as Physical Literacy, autonomous motivation, social competence, social support, flow experiences and sense of meaning emerge as particularly promising areas for further investigation.

Finally, the results underscore the importance of reducing inequalities related to gender, socioeconomic context and access to participation opportunities. Creating more inclusive educational environments that actively promote participation should be a priority for researchers, educators and policy-makers.

## Conclusion

The studies gathered in this Research Topic consistently demonstrate that Physical Education and physical activity play a meaningful role in promoting life satisfaction and well-being among adolescents and young adults. However, the principal contribution of this collection is to show that these benefits do not arise solely from physical practice *per se*, but emerge from a complex interaction of motivational, psychological, social and contextual mechanisms.

Physical Education appears as a privileged context for fostering Physical Literacy, autonomous motivation and behavioural intentions conducive to adopting active lifestyles. At the same time, physical activity contributes to mental health by strengthening interpersonal relationships, social support, positive experiences and the construction of personal meaning.

In sum, the findings of this collection reinforce the need to understand Physical Education and physical activity as instruments of holistic human development. When supported by favourable family, school and social contexts, they can make a substantial contribution to shaping healthier, more resilient, motivated and life-satisfied young people.
